# The Pension Bureau and the G. A. R.

**Published:** 1902-11

**Authors:** 


**A Monthly Review of Medicine and Surgery.**

*EDITOR:*

WILLIAM WARREN POTTER, M. D.

All communications, whether of a literary or business nature, books for review and exchanges should be addressed to the editor: 284 FRANKLIN STREET, BUFFALO, N. Y.

VOL. XLII.—LVIII. NOVEMBER, 1902. NO. 4.

### The Pension Bureau and the G. A. R.

THE JOURNAL for October published an address delivered by Dr. J. F. Raub, medical referee of the Pension Bureau, in which the author discussed methods for improving the medical service connected with that office. The paper referred to was read at Saratoga, N. Y., June 9, 1902, at the first annual meeting of the National Association of United States Pension Examining Surgeons. We invite the thoughtful attention of the medical profession to the subject dealt with by Dr. Raub because we deem it an important one. It is a topic, too, that tells of evils which need the influence of the profession to correct.

It appears that there are about 75 medical officers employed in the bureau at Washington, and 4,500 examining surgeons are distributed throughout the several states. In the cities and larger centers they are grouped in boards, consisting each of three members; in the remote regions single examining surgeons perform the duty. These physicians are appointed by the commissioner of pensions upon the recommendation, generally speaking we believe, of members of congress or of other recognised reliable sponsors. This, in brief, is the organisation; now we will consider for a moment the plaint of the Grand Army of the Republic.

The committee appointed by this organisation of veterans at its annual meeting held in 1901, made its report at the recent encampment held at Washington. It is full of acidity in its arraignment of the Pension Bureau, and next after former commissioner Evans its blows are aimed at the head of the medical service. The complaint is that soldiers entitled under the laws were prevented from getting their just dues by the rulings or orders of Commissioner Evans and the restricted or narrow action of Medical Referee Raub.

The following is an extract from the report on this latter point:

> We are convinced that scores of claims are rejected every day that should be allowed.
>
> The "dead line" or place of execution of the veteran's claim was found in the medical division of the bureau, where unlimited discretion seems to be vested to ignore the reports and ratings of examining surgeons and to minimize the soldiers' disabilities.
>
> We find that the bureau has been exceedingly technical, narrow and harsh in the application of rules of evidence.

To every intelligent man the statements quoted will appear most absurd. Both Mr. Evans and Dr. Raub are veteran soldiers of honorable record, and would indignantly resent any charge even of desire to prevent other veteran soldiers from obtaining their lawful rights under the pension laws. Both are honorable men and would equally defend those dearly-bought rights by preventing, if in their power, the conferring of these privileges in an unlawful manner.

These officers have been displaced, whether because of these attacks of the G.A.R., we know not. Mr. Evans resigned last spring and left the bureau for a higher place in the government service, while Dr. Raub has been relegated to a subordinate place in the medical division. All this happened previous to the Washington Encampment, hence it is difficult to understand the force and import of the encampment's action, even from the viewpoint of the G. A. R. Perhaps it was designed as a note of warning to the new régime,—as a club with which to hold the new officials down to the ideas of the leaders of the soldiers' organisation.

If this be its purpose it is a weak invention, for the present heads of the Pension Bureau and its divisions, like their predecessors, must adhere to the law as interpreted by the Secretary of the Interior, who is the responsible head. It is not out of place to consult, for a moment, the annual report of the Commissioner of Pensions for the fiscal year ended June 30, 1902, which contains some interesting figures. It shows that the loss from the pension rolls by death, marriage, legal limitations, failure to claim and for other causes, was 42,241 in one year. In the same time there was a gain by acts of the Pension Bureau and by special acts of Congress of 43,952, so that there was a net gain on the pension rolls of 1,711. The compiler of the report also estimates the death-rate among the pensioners for the coming year "at about 40,000," and he looks for a reduction of the list "from other causes" by 6,000. The lists which accompany the report show that 738,809 soldiers and 260,637 widows and dependants are receiving pensions. The amount paid to pensioners for 1902 is given at $137,405,268. In 1901 this amount was $138,531,-484. In 1900 the total pension payments were $138,462,131. The pension system since the beginning of the government has cost the vast sum of $2,992,509,019.37. This aggregate is exclusive of the establishment of the soldiers' homes. Surely, the republic is not ungrateful!

In Dr. Raub's paper he discusses calmly and intelligently the difficulties his division encounters from imperfect work done in many instances by incompetent men. He also points out very clearly that properly constructed reports based upon scientific examinations, would serve to clear the pension bureau of many unadjusted claims to the relief of deserving soldiers. If the Grand Army of the Republic would interest itself in aiding to procure the appointment of competent examining surgeons, instead of wasting its energies in attacking the competent officers of the bureau it would again render the country most excellent service. We have already said this is a subject in which the medical profession is interested; or, if not it ought to be. Dr. Raub has done his duty in fearlessly pointing out the difficulties under which the medical division of the pension bureau labors, for which he deserves the thanks of every educated physician.

Let us hope the National Association of Examining Surgeons will become a factor of importance in stimulating better work and in improving the *esprit de corps* of the pension medical service. It deserves the encouragement of the solid ranks of an intelligent profession along these lines.

